# [Retracted] Effects of negative-pressure wound therapy combined with microplasma on treating wounds of ulcer and the expression of heat shock protein 90

**DOI:** 10.3892/etm.2025.12881

**Published:** 2025-05-12

**Authors:** Zhihong Li, Qihong Wang, Wenxin Mi, Mei Han, Fei Gao, Guangyan Niu, Yindong Ma

Exp Ther Med 13:2211–2216, 2017; DOI: 10.3892/etm.2017.4266

Following the publication of the above article, a concerned reader drew to the Editor’s attention that, regarding the CD34 immunohistochemical staining experiments shown in Fig. 2 on p. 2214, there were potential anomalies associated with the data shown in all six panels; essentially, patternings of subgroups of cells within the images were very similar, also appearing in matching positions comparing across various of the figure panels, to the extent that it was difficult to conceive of the similarities as being coincidental. Moreover, concerning the results for the blood perfusion experiments shown in Fig. 1, two pairs of the data panels were strikingly similar, suggesting that these data were derived from the same original source.

After having conducted an internal investigation of the data in this paper, in addition to the incorrect assembly of Fig. 1, the Editor of *Experimental and Therapeutic Medicine* has judged that the potentially anomalous presentation of the strikingly similar groupings of cells in Fig. 2 was too extensive that these features could have been easily attributed to coincidence, Therefore, the Editor has decided that this article should be retracted from the publication on the grounds of an overall lack of confidence in the data. The authors were asked for an explanation to account for these concerns, but the Editorial Office did not receive a reply. The Editor sincerely apologizes to the readership for any incovenience caused, and we thank the reader for bringing this matter to our attention.

